# A 3D Microfluidic Paper-Based Analytical Device with Smartphone-Based Colorimetric Readout for Phosphate Sensing

**DOI:** 10.3390/s26010335

**Published:** 2026-01-04

**Authors:** Jose Manuel Graña-Dosantos, Francisco Pena-Pereira, Carlos Bendicho, Inmaculada de la Calle

**Affiliations:** Centro de Investigación Mariña, Departamento de Química Analítica e Alimentaria, Grupo QA2, Edificio CC Experimentais, Universidade de Vigo, Campus de Vigo, As Lagoas, Marcosende, 36310 Vigo, Spain; jose.manuel.grana.dosantos@alumnos.uvigo.es (J.M.G.-D.); fjpena@uvigo.gal (F.P.-P.); bendicho@uvigo.gal (C.B.)

**Keywords:** phosphate, orthophosphate, paper-based microfluidic devices, smartphone-based detection, water and soil analysis

## Abstract

In this work, a 3D microfluidic paper-based analytical device (3D-µPAD) was developed for the smartphone-based colorimetric determination of phosphate in environmental samples. The assay relied on the formation of a blue-colored product (molybdenum blue) in the detection area of the 3D-µPAD upon reduction of the heteropolyacid H_3_PMo_12_O_40_ formed in the presence of phosphate. A number of experimental parameters were optimized, including geometric aspects of 3D-µPADs, digitization and image processing conditions, the amount of chemicals deposited in specific areas of the 3D-µPAD, and the reaction time. In addition, the stability of the device was evaluated at three different storage temperatures. Under optimal conditions, the working range was found to be from 4 to 25 mg P/L (12–77 mg PO_4_^−3^/L). The limits of detection (LOD) and quantification (LOQ) were 0.015 mg P/L and 0.05 mg P/L, respectively. The repeatability and intermediate precision of a 5 mg P/L standard were 4.8% and 7.1%, respectively. The proposed colorimetric assay has been successfully applied to phosphorous determination in various waters, soils, and sediments, obtaining recoveries in the range of 94 to 107%. The ready-to-use 3D-µPAD showed a greener profile than the standard method for phosphate determination, being affordable, easy-to-use, and suitable for citizen science applications.

## 1. Introduction

Phosphorus (P) is an essential nutrient for the growing of plants which significantly affects the agriculture and livestock [1]. The natural cycle for this nutrient has been changed through the years due to the human action [2]. The rise in P levels is mainly attributed to urban discharges (detergents) and industrial residues, and the application of inorganic fertilizer, infiltrating through soil into groundwater or washed down by rain, and the subsequent transport to diverse water bodies (water reservoir, aquifer water, lakes and rivers, seas, and oceans) [3]. The determination of phosphate in environmental samples is of paramount relevance due to its contribution to eutrophication, a process associated with algae proliferation, which is harmful to ecosystems and human health [4].

Due to its adverse effects in the environment, the European Union has recently updated and lowered the total phosphorus discharge limits in urban wastewater from 2 mg P/L for towns with 10,000 and 100,000 citizens, and 1 mg P/L for larger cities (more than 100,000 citizens) (Directive 91/271) [5] to 0.5 mg P/L (<150,000 citizens) and 0.7 mg P/L (≥150,000 citizens) (Directive 2024/3019) [6].

The American Public Health Association (APHA) describes three standardized methods for the UV-vis spectrophotometric determination of phosphate in drinking water and wastewater (methods 4500P-C, D and E) based on the formation of heteropolyacids [7], with the 4500-P E method being widely applied due to its higher sensitivity.

The ascorbic acid method is commonly employed for soil phosphate determination after a sample preparation step. In this vein, the official Spanish method involves the extraction of soluble phosphate in sodium hydrogencarbonate (0.5 M) [8]. In general, the traditional methods require large amounts of reagents to perform both the extraction and the formation of the colored product for analysis. Alternative methods have also been performed for the determination of phosphates, including ion chromatography with conductivity detection [9], fluorescence [10], electroanalysis [11], inductively coupled plasma optical emission (ICP-OES) and inductively coupled plasma mass spectrometry (ICP-MS) [12], even though they mostly require centralized equipment with high costs of acquisition and maintenance. In the last years, non-instrumental detection approaches have been proposed as affordable and suitable alternatives for on-site optical detection [13,14,15]. Several recent miniaturized approaches developed for phosphate determination were based on the use of a metal–organic framework (MOF), for instance by bimodal detection (fluorescent and colorimetric) [16], by a smartphone colorimetric detection using both an array with multi-functionalized MOF for eight physiological phosphate species [17], and by using a bimetallic Ce-Fe-MOF [18] for biological and environmental samples. Non-instrumental detection is especially suited to miniaturized systems like microfluidic paper-based analytical devices (µPADs), where cellulose substrates enable sample transportation by capillarity without the need of external pumps to perform chemical reactions [19,20,21].

A range of PADs and µPADs combined with colorimetric analysis have been developed for phosphate detection (Table S1 of the Supplementary Material) [1,4,22,23,24,25,26,27,28,29,30,31,32,33,34,35], being mainly applied to the analysis of water samples, with few contributions being applied to more complex matrices, such as soil extracts, toothpaste or fertilizer samples [4,29,30,33,34]. A wide variety of PADs were designed from the simplest circular spot test [1,23] to bidimensional 2D-µPADs [4,24,30], tridimensional 3D-µPADs [4,22,25,29], paper test strips [26,27,28], dip-PADs [31], 3D-µPADs [34], multiplex 3D-µPADs for three analytes but based on distance for phosphate [32,33], and distance-based µPADs [35]. These approaches offer advantages over classical methods in terms of cost and portability, although challenges remain.

The fabrication of PADs and µPADs often involves time-consuming (20 min–3 h) and labor-intensive steps. For instance, these steps were the following: (i) thermal treatment for hydrophobization (150 °C, 30 min) [4,22], to cure the polydimethylsiloxane (PDMS) (80 °C, 30 min) [23] or air drying at room temperature of the PAD after screen-printed epoxy resin (3 h) [29]; (ii) air drying of reagents (ascorbic acid, molybdate, H_2_SO_4_, and antimony) in the PAD or µPAD (10 min) [29], air drying of reagents (30 min) with additional overnight drying (−20 °C) [25], or air drying of four separate aliquots of 3 µL of ascorbic acid (4 × 20 min, 80 min in total) [24], or three aliquots of 1.2 µL of ascorbic acid solution [34]; (iii) the immobilization of the colorimetric reagents in the paper strip (2 h undisturbed + 30 min at 60 °C) [26], the modification of the paper strips with ascorbic acid and potassium and antimony tartrate (5 min for paper saturation and 2 h of air drying) [27] or air drying of reagents in the hydrophilic channels chemically modified (1 h) [33]; and (iv) the addition of the sample, followed by blow air drying, addition of the necessary reagents (molybdate, H_2_SO_4_, tartaric acid, and ascorbic acid), and the corresponding air drying at the moment of the analysis (6 min) [1].

In many works, highly acidic solutions were used, like sulfuric acid (6.6 M [4], 6 M [34]), nitric acid (6 M) [32], or even p-toluene sulfonic acid (7.5 M) [33], that may compromise paper integrity due to the hydrolysis of cellulose, especially for sulfuric acid.

Additional strategies were developed for enhancing the stability that further complicate their preparation. For instance, several works used interleaving sheets of cellulose acetate or polytetrafluorethylene to avoid the contact between the reagent zones [4,22], or laminated layers to avoid evaporation of reagents that required the punching of sample holes (with a scalpel) before sample addition [24,25]; even lamination was performed at 80 °C [25] or 125 °C [33]. Double-sided adhesive and transparent film were also used [31].

Additionally, device disassembly and unfolding of 3D-µPADs prior to phosphate detection hinders their operation [34]. Moreover, dip-PADs demand strict control of sample dipping times or sequential reagent additions immediately before analysis to improve the precision [1]. In spot test devices, the sample, molybdate and tartaric acid in sulfuric acid, and ascorbic acid were sequentially added and submitted to blow drying after each addition and before detection just at the moment of the analysis [1].

In fact, only some ready-to-use µPADs modified with all required reagents have been reported in the literature for phosphate determination, whereas the addition of chemicals at the time of analysis is mandatory in several PADs. This fact represents a constraint for the use of PADs, especially in community-based monitoring studies, requiring, e.g., (i) the addition of Mo reagent to the PAD immediately before the sample [24] or immersion of the modified test strip onto a molybdate acidic solution immediately before immersion onto the sample [26]; (ii) the mixture in a vial of sulfuric acid, molybdenum reagent, and the sample at the point of need before the immersion of the paper strip (previously modified with ascorbic acid and antimony reagent) on the vial [27]; (iii) the addition of acidic reagents to the paper strip before immersion in the sample [28]; or (iv) simultaneous addition of sample and reagents in two different zones of a dumbbell-form PAD [30].

Devices preloaded with the reagents required for P determination, which can be stored until analysis and transported for in situ analysis, would be highly desirable. However, several PADs and µPADs lack preloaded reagents for the colorimetric assay, thus requiring the addition of reagents (e.g., sulfuric acid, Mo reagent, or both [1,24,26,27,28,30] to the cellulose substrate immediately prior to the analysis, thus hindering their suitability for field analysis. Lastly, it would be of great interest to develop a PAD or µPAD that is fast to prepare, with a not very high concentration of immobilized acid that allowed the detection of phosphate in environmental samples at low levels of concentration and covered the determination of the anion in a wide range of concentrations. In addition, reagents consumption and the extent of analytical information can be further optimized, and the stability and selectivity of µPADs requires thorough evaluations.

Thus, in this work, a non-instrumental analysis method for the determination of phosphate in different samples using cost-efficient preloaded 3D-µPADs and smartphone readout was developed. This 3D-µPAD can be easily and rapidly prepared, avoiding the use of highly concentrated acids, and can be stored with all reagents preloaded in separate layers, remaining ready for sample introduction, analysis, and real-time detection without a disassembly step. Moreover, to the best of our knowledge, no reports describe three-layer origami 3D-µPADs for phosphate determination in which the microfluidic transport (both longitudinal and transverse) occurs across all three layers. The devised 3D-µPAD enables the simultaneous processing of a blank and a standard or a sample, both in duplicate, since the introduced target solution is split into two channels, thus minimizing potential inconsistencies associated with the luminosity changes and digitization step. Experimental parameters were systematically optimized, including geometric aspects of 3D-µPADs, digitization and image processing, the amount of chemicals added to the 3D-µPAD, and the reaction time. The shelf life of the device was evaluated under different storage conditions, and the effect of incorporating reagents such as ethylene glycol and p-toluenesulfonic acid into preloaded 3D-µPADs was also assessed. An exhaustive interference study was performed, and the applicability of the 3D-µPAD was evaluated with samples of increasing complexity, including waters, soil extracts (P-NaHCO_3_-extractable), a sediment extract (P-NaOH-extractable), and a sewage sludge digestate.

## 2. Materials and Methods

### 2.1. Reagents and Materials

All reagents were of analytical grade and solutions were prepared in ultrapure water. A water purification Millipore Sigma Simplicity (Millipore, Iberian, Madrid, Spain) was used throughout. The reagents used for phosphate determination were dihydrogen potassium phosphate (Merck, Darmstadt, Germany), ammonium molybdate (Scharlau Chemie S.A, Barcelona, Spain), potassium, and antimony tartrate trihydrate (Sigma-Aldrich, St. Louis, MO, USA), sulfuric acid (Merck), L-ascorbic acid (Sigma-Aldrich), sodium bicarbonate (Carlo Erba, Milan, Italy), sodium hydroxide (AnalaR Normapur, Leuven, Belgium).

The reagents used for the interferences studies included an atomic absorption standard of 1 g/L of Si as ammonium hexafluorosilicate (VWR Chemicals, Radnor, PA, USA) and 1 g/L As(V) prepared from disodium hydrogen arsenate heptahydrate (Na_2_HAsO_4_·7H_2_O) (Panreac, Barcelona, Spain). Solutions of 1 g/L of the following reagents were prepared: Na_2_S·3H_2_O, KCl and H_3_BO_3_ (VWR Prolabo, Leuven, Belgium), K_2_CrO_4_, FeCl_3_·6H_2_O, NaNO_2_ and Na_2_CO_3_ (Panreac), NaCl (VWR Chemicals), CuCl_2_·2H_2_O and NH_4_VO_3_ (ammonium monovanadate) (Merck), CaCl_2_·2H_2_O (Fisher Chemical, Pittsburgh, PA, USA), KNO_3_, (Probus, Badalona, Spain), NH_4_Cl (Acros Organics, Geel, Belgium), NaHCO_3_ (Carlo-Erba, Milan, Italy) and Na_2_SO_4_ (Sigma-Aldrich).

The following reagents were used for the preparation of synthetic wastewater samples [36,37]: sodium sulfate (Carlo Erba), potassium nitrate, copper (II) sulphate and ammonium nitrate (Probus), sodium chloride (VWR Chemicals), potassium dichromate and zinc nitrate hexahydrate (Scharlau Chemie S.A), calcium chloride (Prolab, Briare Le Canal, France), dihydrogen potassium phosphate dehydrate (Merck) and tin (II) chloride (Prolabo, Fontenay-sous-Bois, France).

Ethylene glycol (Fluka, Buchs, Switzerland) and toluene-4 sulfonic acid monohydrate (p-toluene sulfonic acid, pTsOH, C_7_H_18_O_3_S) (Supelco, Darmstadt, Germany) were used in stability studies involving 3D-µPADs.

### 2.2. Apparatus and Materials

A Xerox ColorQube 8580 wax printer (Rochester, NY, USA) and a force air drying oven (Lbx Instruments, Barcelona, Spain) were used for the fabrication of µPADs, using Whatman No. 1 filter paper (Maidstone, Kent, UK) as substrate. A homemade methacrylate holder designed and described in a previous work [38] and a portable LED lightbox PULUZ (Shenzhen PULUZ Technology Limited, Shenzhen, China), equipped with 20 LEDs, and a smartphone Redmi Note 8 Pro (Xiaomi, Beijing, China) and Samsung Galaxy A53 (Suwon, Republic of Korea) were used for the digitization of the µPADs. The free software ImageJ (version 1.54g) was used for the processing of images and data acquisition [39].

An orbital mechanical shaker Fisherbrand (Heathrow Scientific, Vernon Hills, IL, USA) and Whatman filters No. 40 (Maidstone, Kent, UK) were employed for the sample preparation of soil samples. Magnetic agitation stirrer mix 8 XL (2mag AG, Munich, Germany) was used for the extraction of P from the sediment sample. A microwave digestion system Multiwave 3000 Oven (Anton Paar, Graz, Austria) was used to digest the sewage sludge, and 0.45 μm cellulose nitrate filters (Sartorious, Göttingen, Germany) were employed with a Pyrex^®^ Vacuum filtration system for the filtration of seawater samples.

### 2.3. Samples and Sample Preparation

Several water samples of increasing complexity were analyzed with the proposed assay, including a bottled drinking water, a river water (Lagares river, Vigo, Spain), a seawater sample (Tombo do Gato beach, Vigo, Spain), and two synthetic wastewater samples prepared containing phosphate [36,37]. Sample pretreatment was not required for the analysis of water samples with the exception of the seawater sample, which was filtered through nitrate cellulose filters (0.45 μm) to remove potential suspension solids.

Additionally, three solid samples were analyzed, including two CRMs, namely sediment (BCR 684) and sewage sludge (CRM 029), and two soil samples. These samples were dried at 105 °C for 24 h before analysis.

Two agricultural soil samples with origins in different areas of Cataluña (Spain) and preceding from an interlaboratory exercise were analyzed by both the proposed assay and the official UV-vis spectrophotometric method [8]. A sample preparation procedure described in the official method was used in both cases. In brief, 5 g of sample was introduced in a polyethylene bottle and mixed with 100 mL of a 0.5 M sodium hydrogencarbonate (pH 8.5) [7,8]. Extraction was performed using a mechanical orbital shaker (200 rpm, 30 min). Afterwards, the extract was filtered through Whatman No. 40 filter paper for further analysis.

The CRM 029 Trace Metals—Sewage Sludge 2 (Supelco, Sigma-Aldrich) was used to evaluate the total P content. Then, 0.1 g of CRM 029 was digested in a microwave oven with 4 mL HNO_3_ (65% *w*/*w*), 2 mL H_2_O_2_ (30% *w*/*w*), and 1 mL HCl (37% *w*/*w*), diluted to 25 mL and finally diluted 1:4 in ultrapure water before P determination.

BCR 685 extractable phosphorus in sediment (Institute for Reference Materials and Measurements, IRMM, Geel, Belgium) was used to evaluate the P extractable in NaOH 1 M. Then, 0.4 g of BCR 685 underwent extraction for 16 h by magnetic stirring in 20 mL NaOH 1 M followed by centrifugation at 5200 rpm for 15 min and diluted 1:2 before analysis.

### 2.4. Design and Fabrication of 3D-µPADs for the Determination of Phosphates

**Figure 1:**
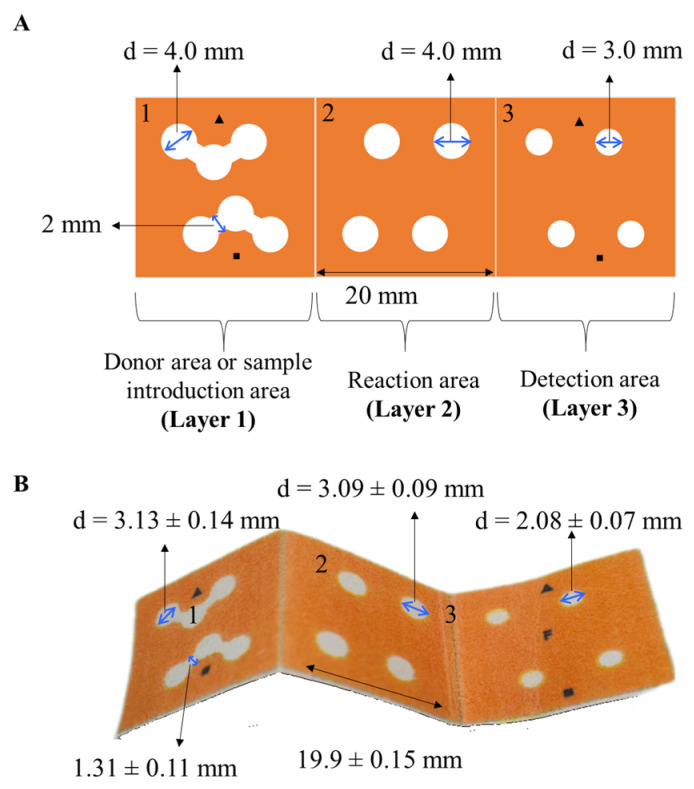
(**A**) Schematic design of the 3D-µPAD. (**B**) Image of the folded 3D-µPAD after thermal treatment.

The 3D-µPAD design (Figure 1) was wax-printed onto cellulose substrates and subsequently subject to thermal treatment in a laboratory oven (130 °C, 2 min) to create hydrophobic patterns that define hydrophilic channels and reservoirs. Each 3D-µPAD was cut before use. The dimensions of the reservoirs and channels before and after wax printing are indicated in Figure 1.

The 3D-µPAD (60 mm × 20 mm) comprises three independent squared areas (20 mm × 20 mm each zone). The first layer (1) corresponds to the donor area or sample introduction zone, where both blank and standard or sample solution are simultaneously added. This layer consists of two identical parts, separated through the symmetrical axis of the 3D-µPAD. Each part contains a circular inlet (d = 4 mm) connected by two fluidic channels of 2 mm wide to two circular areas (d = 4 mm). The introduction of the blank and the standard/sample in this layer is performed through the central circular areas. This design thus enables the processing of a blank and a standard or sample in duplicate under identical conditions.

The intermediate layer (2) corresponds to the reaction area, composed of four identical circular areas (d = 4 mm) aligned with the hydrophilic areas of upper and down layers. A volume of 5 µL of 0.1 M ascorbic acid solution (i.e., 88.1 µg immobilized in the reservoir) is added in the four hydrophilic areas of this layer.

**Figure 2:**
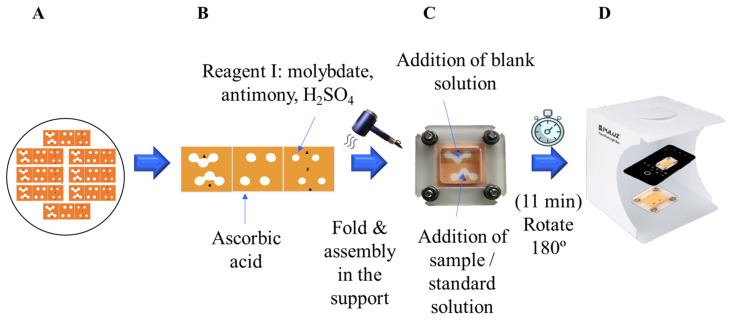
Design, fabrication of 3D-µPAD, and experimental procedure for the determination of phosphates. (**A**) Design of the 3D-µPAD. (**B**) Immobilization of reagents (ascorbic acid and reagent I) and drying. (**C**) Assembly of the 3D-µPAD in the methacrylate holder and addition of blank and sample/standard solution. (**D**) Rotate 180°, digitization after 11 min.

The third layer (3) corresponds to the detection area, composed of four identical circular areas (d = 3 mm) where 3 µL of the reagent I (composed of 20 mM ammonium molybdate, 1.23 M sulfuric acid, and 0.8 mM potassium and antimony tartrate trihydrate solutions) are added, i.e., 73 µg of ammonium molybdate, 362.2 µg sulfuric acid, and 1.6 µg potassium and antimony tartrate trihydrate immobilized in the reservoir. Figure 2 summarizes the steps involved in the fabrication of the 3D-µPAD for phosphate determination. 

Finally, the 3D-µPADs are dried by using a hair drier and stored in polyethylene bags in the freezer before analysis or prepared on the same day of use.

### 2.5. Reaction and Transport Mechanism in the 3D-µPAD

The method employed in this work (the molybdenum blue method) is based on the formation of molybdophosphoric acid (12-MPA) (H_3_PMo(VI)_12_O_40_) (reaction 1) in an acidic medium and subsequent reduction with ascorbic acid to obtain the blue-colored phosphomolybdenum blue complex (PMB) ([H_4_PMo(VI)_8_Mo(V)_4_O_40_]^−3^) (reaction 2) [40]. The reaction is catalyzed by antimony and potassium tartrate.

PO_4_^3−^ + 12 MoO_4_^2−^ + 27 H^+^ → H_3_PMo(VI)_12_O_40_ + 12 H_2_O (1)

H_3_PMo(VI)_12_O_40_ + Ascorbic acid → [H_4_PMo(VI)_8_Mo(V)_4_O_40_]^3−^ (2)

Due to the incompatibility of reagents (the molybdate/H_2_SO_4_/Sb/ascorbic acid mixture in the spectrophotometric method yields a blue color even in the absence of phosphate probably due to the reduction of Mo(VI) to Mo(V) by ascorbic acid after 24 h from the preparation [4]), ascorbic acid was added to the second layer of the µPAD, whereas the reagent I (i.e., molybdate, H_2_SO_4_, and antimony and potassium tartrate) was added to the third layer. After drying using a hair drier (4–5 min at room temperature), 3D-µPADs were stored unfolded in plastic bags to avoid contact between the different zones.

The prepared 3D-µPAD is folded as a Z-form (or accordion fold, i.e., layer 1 in contact with layer 2, and layer 2 in contact with layer 3), placed in the lab-made methacrylate holder (35 mm × 35 mm) adjusted with four screws, as previously described [38] and depicted in Figure 2.

The system is placed with layer 1 (containing the sample introduction zones and reservoirs) upwards. Then, 15 µL of target solutions (blank, standard, or sample) are deposited onto the two hydrophilic donor/inlets’ central circular reservoirs in the sample introduction area. Then, each solution is divided into two hydrophilic channels (allowing duplicate analysis) by longitudinal capillarity (the fluid flows along the length of the channel). Then, these solutions move by transversal capillarity (the fluid flows perpendicular to the hydrophilic area) to the layer below containing the ascorbic acid (layer 2) and to the layer below containing the reagent I (layer 3). Thus, the reaction of the molybdenum blue method occurred, resulting in the appearance of a blue-colored product in the detection area.

The folding and assembly of the 3D-µPAD in the methacrylate holder guarantee the pattern alignment between the three layers and reservoirs, ensuring the proper connection of the four sample reservoirs (layer 1) with the reaction areas (layer 2) and the detection areas (layer 3). Thus, it allowed the fluid to flow in both the vertical and the horizontal directions on adjacent layers avoiding sample leak.

Additionally, this device provides more reproducible interlayer contact, ensuring the homogenous formation of the colored product in the detection area and allowing the direct detection of the assembled 3D-µPAD without having to unscrew, disassemble, and unfold the 3D-µPAD for smartphone detection.

### 2.6. Experimental Procedure for the Determination of Phosphates and Data Processing

After 11 min of reaction time, the 3D-µPAD was turned over and placed in the portable LED light box with controlled luminosity conditions, and the detection area of the 3D-µPAD digitized using the smartphone camera (digitization conditions: ISO 1600, EV +1.7 and WB 7000 K) positioned at a distance of 14 cm from the 3D-µPAD. The obtained digital images were then processed using the ImageJ software. The color intensity difference in the red channel of the RGB color space (i.e., ∆Ic(R) = Ic Blank (R) − Ic target (R)) was used for quantitative purposes.

## 3. Results and Discussion

### 3.1. Design Optimization and Configuration of the 3D-µPAD for Phosphate Determination

A 3D-µPAD was designed in this work to overcome certain limitations observed in previous PADs and µPADs for phosphate determination. The design, depicted in Figure 1, included two donor inlets that facilitated performing the measurements of the blank and the standard or sample in a simultaneous way, thus minimizing possible side effects associated with variations in the luminosity conditions that could affect the analytical response. In addition, two reservoirs connected with each of the donor inlets through hydrophilic channels were included to allow the performance of replicated analysis. The 3D-µPAD was thus designed with two identical parts for the processing of each solution.

**Figure 3:**
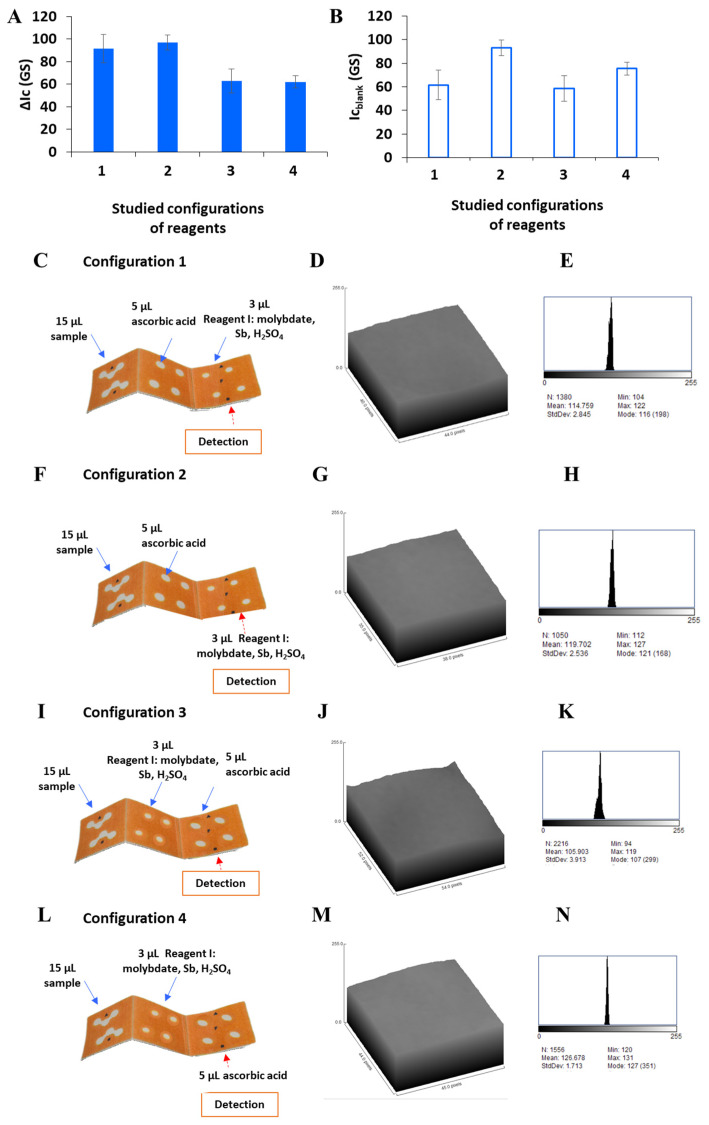
Evaluation of different configurations depending on the preparation of the 3D-µPAD. (**A**) Grayscale-corrected signal for different configurations. (**B**) Grayscale signal for blanks obtained with the four different configurations. (**C**,**F**,**I**,**L**) show the scheme of the different configurations. (**D**,**G**,**J**,**M**) show the surface plots of the different configurations. (**E**,**H**,**K**,**N**) show the histograms of the different configurations. (Blue arrows represent the addition of reagents in the upper layer, and red arrows represent the addition of reagents in the reversed layer and detection area.).

The lack of compatibility of the chemicals required for analysis was overcome by physically immobilizing them in separate reservoirs of the intermediate layer and detection layer of the 3D-µPAD, taking advantage of the principles of origami. Different configurations were evaluated for optimal performance (Figure 3).

Specifically, it was experimentally verified that the position where the reagent I and the reductant were immobilized in the 3D-µPAD in relation to the addition of blank or standard and the detection zone presents a significant effect on the color intensity and the homogeneity of the product in the detection zone (comparing surface plots and histograms). In configuration 1, the sample, the reducing agent, and the reagent I were immobilized on the same side of the paper, each one in the layers 1, 2, and 3, respectively; but the detection was performed on the opposite side of the paper after inversion of the assembled 3D-µPAD. In configuration 2, the reagent I is immobilized on the opposite side of the paper, where the detection step is performed. In configurations 3 and 4, the reagent I was added to layer 2 near to the sample and on the same side of the paper. In configuration 3, ascorbic acid was immobilized in layer 3 on the same side of the paper, and the detection was performed on the opposite side of the paper. In configuration 4, both the addition of ascorbic acid and the detection step were performed on the opposite side of the paper of sample and reagent I.

As shown in Figure 3, the best results were obtained when the reducing agent (ascorbic acid) was immobilized in the intermediate layer, and the reagent I, composed of ammonium heptamolybdate, sulfuric acid solution, and potassium and antimony tartrate, was immobilized in the detection layer of the 3D-µPAD (configurations 1 and 2) both in the same or opposite face of the paper. Additionally, configurations 2 and 4, where the reagent I or ascorbic acid was immobilized on the same face of the paper where detection takes place, produced a higher blank signal (Figure 3B). Additionally, the surface plots show less homogeneity and lower precision for configuration 3 (Figure 3J) and a wider color intensity range from 94 to 119 (Figure 3K). Thus, configuration 1 was used for further experiments. Additionally, this configuration is more convenient, since the reagents can be simultaneously immobilized and dried, with the consequent time saving.

**Figure 4:**
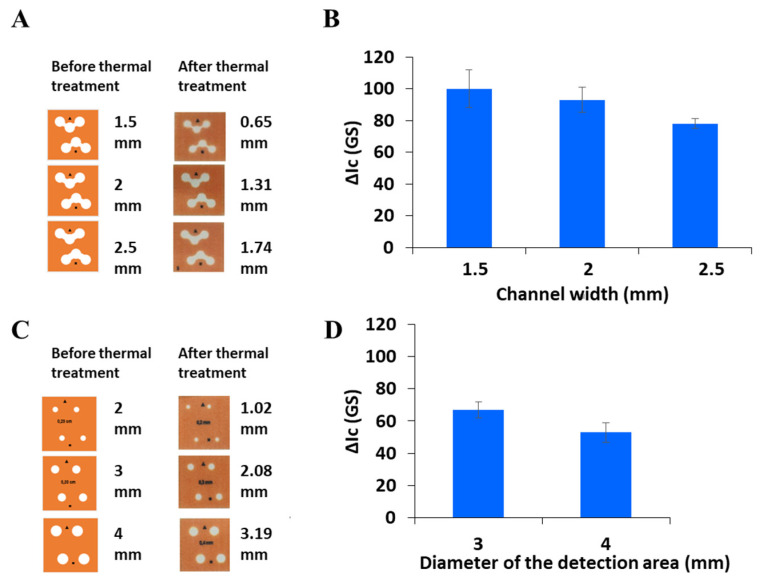
Study of the parameters involved in the design. (**A**) Images of 3D-µPADs before and after thermal treatment of different channel widths of the sample zone. (**B**) Grayscale-corrected signal for different channel widths of the sample zone. (**C**) Images of 3D-µPADs before and after thermal treatment of different diameters of reservoir detection area. (**D**) Grayscale-corrected signal for different diameters of reservoir detection area.

The width of the hydrophilic channels and the diameter of the reservoirs of the detection zone were subsequently evaluated, bearing in mind the importance of these geometric parameters on the analytical performance [38]. The hydrophilic channels must ensure an efficient capillary-driven fluid transport of the blank, standard, or sample solutions to the corresponding reservoirs, thus avoiding potential flow blockage. It is well established that the thermal treatment required to create hydrophobic patterns on µPADs after wax printing leads to channels and reservoirs with reduced dimensions [38,41]. Thus, both the width of the sample zone channels and the diameter of the detection zone reservoirs were evaluated, and the results are shown in Figure 4. Firstly, 3D-µPADs with three different initial channel widths (namely, 1.5 mm, 2.0 mm, and 2.5 mm) in the donor area were tested (Figure 4A,B). The width of the channels after thermal treatment was measured and found to be 0.65 ± 0.14 mm, 1.31 ± 0.11 mm, and 1.74 ± 0.07 mm, respectively. As expected, the best results were obtained when the narrower channels were used, since they ensured a more efficient transport of phosphate ions to the detection zones. However, the use of 3D-µPADs with channels of 1.5 mm width (0.65 ± 0.14 mm width after thermal treatment) supposed the flow blockage of solutions in a non-negligible number of cases. According to this, channels of 2.0 mm of initial width (1.31 ± 0.11 mm width after thermal treatment) were selected.

Subsequently, the influence of reservoir diameter within the detection zones on the analytical response was assessed. It has been reported in the literature that the use of detection reservoirs with smaller diameter than that of reservoirs present in intermediate layers can enhance the color intensity of the colored product and even improve its homogeneity at the detection area [38]. Thus, detection reservoirs with initial diameters in the range of 2 to 4 mm were evaluated. As commented above, the thermal treatment of 3D-µPADs leads to a reduction in the diameter of the hydrophilic detection zones (the initial diameters of 2 mm, 3 mm, and 4 mm were reduced to 1.02 ± 0.11 mm, 2.08 ± 0.07 mm, and 3.19 ± 0.14 mm, respectively). To maintain a constant mass of reagents in this study, repetitive injection/drying cycles of the reagent I (5 times × 1 µL) were required to prepare the 3D-µPAD with the lowest diameter that was evaluated (i.e., 2 mm), compromising the integrity of the substrate and, thus, impeding the acquisition of an analytical response. The best results were obtained when selecting detection reservoirs with an initial diameter of 3 mm (Figure 4C,D), which also provided a better homogeneity of the colored product.

### 3.2. Digitization and Image Processing Conditions

**Figure 5:**
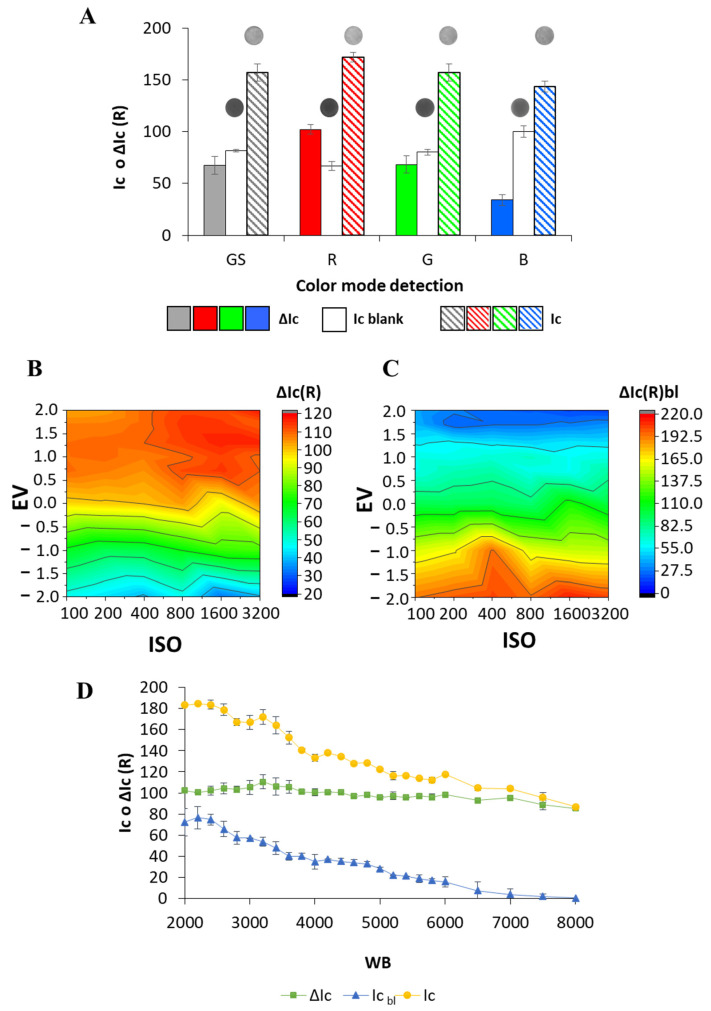
Digitization conditions. (**A**) Effect of the RGB channel. The inset shows the digital images of detection areas of the 3D-µPAD in the absence (blank) and presence of phosphate (standard) obtained at each color channel (R, red; G, green; B, blue and GS, grayscale). The filled bars correspond to the corrected signal obtained for the standard in each color channel. The white bars correspond to the signal of the blank in each color channel. The stripped bars correspond to uncorrected signal of the standard obtained in each color channel. Each color corresponds to each channel: red (red channel), green (green channel), blue (blue channel) and grey (grayscale mode). (**B**) Effect of EV and ISO on the analytical response of a phosphate standard and (**C**) effect of EV and ISO on the analytical response of a blank. (**D**) Effect of the WB.

The digitization and image processing conditions were assessed to obtain the highest sensitivity for the determination of phosphate, while ensuring low values of blanks. Firstly, the effect of the three color channels of the RGB color space on the analytical response was evaluated (Figure 5A). The highest analytical response was achieved when selecting the R channel, which also yielded acceptable blanks. Thus, the red channel was selected for subsequent studies. Subsequently, the digitization parameters of the smartphone camera, namely the light sensitivity (ISO), exposure value (EV), and white balance (WB) were evaluated.

The ISO parameter indicates the sensitivity of the sensor to the light, so that the higher the ISO, the higher the brightness of the images. The EV parameter is a combined parameter that considers both aperture and shutter speed and is related to the amount of light that reaches the sensor of the camera, affecting the clarity or darkness of images. The effect of ISO and EV parameters on the analytical signal in the absence and presence of phosphate was simultaneously evaluated, and the obtained results are shown in Figure 5B,C. As can be deduced from the figure, the highest analytical response (∆Ic) was reached when high values of ISO and EV were selected (Figure 5B). Regarding the blanks, the signal highly depended on the EV, whereas a much lower dependence on the ISO was observed, reaching very low blank signals when an EV of +1.7 and ISO values higher than 200 were used (Figure 5C). Accordingly, the ISO and EV were set at 1600 and +1.7, respectively, since they provided the highest ∆Ic and the lowest blank values.

The WB parameter can also be adjusted for optimal performance, allowing the white color in images to be more natural by adjusting the camera color temperature. This parameter affects the reproduction of colors and is related to the temperature of the image. The WB was studied in the range of 2000–8000 K, and the obtained results are presented in Figure 5D. As can be observed, the WB showed a limited effect on the analytical response (∆Ic) in the evaluated range, whereas the adjustment of the WB parameter showed a paramount effect on the analytical signal of the blank. The decreasing blank signal observed when increasing the WB value can be attributed to the fact that the images take on a bluish hue at low values of WB, thus leading to relatively high color intensities in the absence of analyte. A WB of 7000 K was finally selected to obtain high ∆Ic values with acceptable blanks.

### 3.3. Optimization of Experimental Parameters Affecting the Formation of the Colored Product

Experimental parameters affecting the formation of the colored product were optimized to maximize the sensitivity and homogeneity in the detection zone [42]. Specifically, the amount of ammonium heptamolybdate, catalyst (if needed), and acid solution in the detection zone and the type and amount of reducing agent in the reaction zone of the 3D-µPAD were evaluated and controlled.

**Figure 6:**
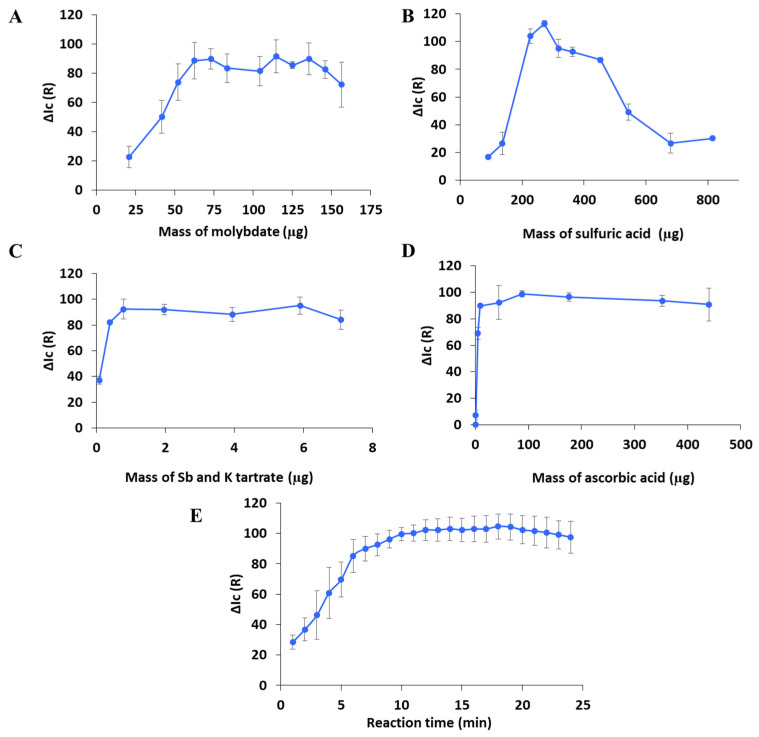
Optimization of chemical parameters. (**A**) Study of the mass of ammonium heptamolybdate, (**B**) sulfuric acid, (**C**) antimony potassium tartrate, and (**D**) ascorbic acid. (**E**) Optimization of the reaction time.

The amount of ammonium heptamolybdate was first optimized, as recommended in the literature [42]. This reagent reacts with phosphate to form 12-MPA and, therefore, controlling the mass of heptamolybdate is essential to ensure a quantitative formation of the product. However, an excessive amount of heptamolybdate may result in the direct reduction of Mo(VI) to Mo(V) [43]. The mass of ammonium heptamolybdate deposited in the detection area was studied in the range 20–160 µg, and the results are presented in Figure 6A. As can be seen, the analytical response (∆Ic) increased by increasing the mass of the reagent up to 60 µg, then reaching a plateau beyond this amount. It should be noted that the use of an amount of heptamolybdate higher than 80 µg resulted in an important increase in the signal of the blank, which can be attributed to the direct reduction of molybdate to Mo(V) [4], resulting in the formation of blue-colored species [42]. According to the above, 73 µg of ammonium heptamolybdate was selected as the optimal value.

The colorimetric determination of phosphate requires a strong acid medium, and sulfuric acid is usually employed with this aim (Method 4500 P-E). In acidic media, molybdate is protonated, then leading to polymerization and the formation of 12-MPA [42]. Different molybdate species can be obtained in aqueous solution depending on the pH, with the 12-MPA being the most known. Additionally, the reduction of the 12-MPA product is pH-dependent. The amount of sulfuric acid deposited in the detection area was studied in the range 90–815 µg, and the obtained results are presented in Figure 6B. The use of a low acid-to-molybdate ratio favors the direct reduction of molybdate, resulting in the formation of a wide variety of blue species of isopolymolybdenum [43] that dramatically increase the blank signal. This effect is observed when using less than 250 µg of sulfuric acid, thus leading to reduced ΔIc values, as shown in Figure 6B. Conversely, the use of large amounts of sulfuric acid results in the degradation of the cellulose paper by hydrolysis, affecting its integrity and hampering the detection step, as described in the literature [25,31]. Additionally, the use of high acid-to-molybdate ratios results in the inhibition of the formation of the blue product [42], as presumably occurs when an amount of sulfuric acid higher than 450 µg was employed. Therefore, an amount of sulfuric acid of 362 µg was selected for subsequent studies.

Sb(III) is commonly used as a catalyst in the reduction of 12-MPA with ascorbic acid, since this reaction is kinetically slow. In the presence of this catalyst, a reduced blue-colored product [PSb_2_Mo_12_O_40_]^−^ (Sb_2_PMB) is formed [44]. The results associated with the effect of the mass of antimony potassium tartrate on the analytical response are shown in Figure 6C. The highest analytical response was achieved with a mass of reagent higher than 0.8 µg. Accordingly, a mass of 1.6 µg of potassium and antimony tartrate trihydrate was selected.

Ascorbic acid is the most used reducing agent for the reduction of 12-MPA, and it was evaluated in this work. As commented above, the reaction involving ascorbic acid is kinetically slow and a catalyst such as Sb(III) is commonly employed. As shown in Figure 6D, the maximum analytical response was obtained when using a mass of ascorbic acid equal to or higher than 88 µg. A mass of ascorbic acid of 88 µg was selected since it provided satisfactory values of ∆Ic using a reasonable amount of reducing agent. Thus, a combination of ascorbic acid and antimony potassium tartrate was selected in this work for the reduction of the 12-MPA.

Finally, the reaction time should ensure the quantitative formation of the 12-MPA and its further reduction. The effect of this variable was evaluated up to 25 min, and the results are provided in Figure 6E. As can be deduced from the figure, the analytical response increased up to ca. 10 min, then reached a plateau. The selection of reaction times longer than 10 min did not result in an enhancement of the analytical response, and the precision was worsened at extended times. Thus, the reaction time was set at 11 min for further experiments.

### 3.4. Assessnebt of the Stability of the 3D-µPAD

**Figure 7:**
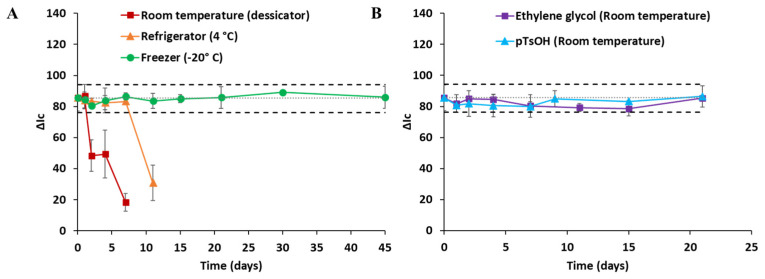
Stability study of the 3D-µPADs stored in different conditions. (**A**) Room temperature, fridge (4 °C), and freezer (−20 °C). (**B**) Room temperature with ethylene glycol and p-TsOH. Dotted lines correspond to ±10 % of the corrected signal of the phosphate standard (15 mg P/L) in initial conditions of fabrication and use of the 3D-µPAD.

The fabrication of µPADs that enable their use with optimal performance even several days after being produced is highly convenient, especially for field analysis, since transporting reagents to the sampling site, filling reservoirs of the µPADs, and subsequent air drying before analysis would be avoided. In this study, the stability of 3D-µPADs was evaluated by storing 3D-µPADs fabricated as described in Section 2.4 under three different conditions before their use, namely room temperature (20 °C), fridge (4 °C), and freezer (−20 °C), and the obtained results are shown in Figure 7A. In this study, it was considered that no significant differences existed in the range ± 10% with respect to the initial conditions of fabrication and use of the 3D-µPAD. Thus, between these limits, it can be considered that the obtained results were comparable between days and with a similar sensitivity. As can be deduced from the figure, the sensitivity of the assay greatly depended on the storage conditions of the 3D-µPAD. Thus, the analytical response was maintained as invariable for at least 45 days when the 3D-µPADs were stored at −20 °C in the freezer. In contrast, the stability of the 3D-µPAD was reduced to 7 days when stored in the refrigerator, presumably due to the autoreduction of Mo species, and this effect was enhanced at room temperature, showing a 50% reduction in sensitivity after 2 days of storage. Bearing in mind the major impact of temperature on stability, it is recommendable to store the 3D-µPADs at −20 °C (in a conventional home freezer) to extend their lifetime (shelf life) before analysis, especially when intended for on-site use. Additionally, previous studies indicate that ethyleneglycol (EG) [24,34] and p-toluenesulfonic acid (pTsOH) [25,31,33] can extend the storage time of PADs and µPADs because EG stabilizes the Mo complex and pTsOH provides acidity and reduces the autoreduction of Mo. In accordance with prior research, we observed that the storage time can be extended to 21 days with 1361 µg of EG (3 µL of the reagent I containing 7.31 M of EG in addition to the other reagents) and 21 days with 1632 µg of pTsOH (3 µL of the reagent I containing 2.86 M of pTsOH instead of sulfuric acid) without refrigeration (Figure 7B). The 3D-µPAD with EG and pTsOH stored after 21 days can be easily broken in the moment of bending. Thus, for on-site and citizen-science applications it is advisable to have a 3D-µPAD preloaded also with EG or pTsOH when storage in a freezer (up to 3 weeks) or refrigerator (up to 7 days) is not feasible.

### 3.5. Evaluation of Potential Interferences

The effect of potential interferences on the analytical response has been evaluated under optimal conditions, and the obtained results are provided in Figures S1 and S2 of the Supplementary Material. Analytical response variations of up to ±10% were not considered significant. Arsenate, chromate, nitrite, sulphide, and silicate are typical interferences of the 4500P-E standardized method [7] and, thus, these anionic species were evaluated with the reported 3D-µPAD. In particular, significant (positive) effects were observed in the presence of As(V) and silicate at concentration levels equal or higher than 2 mg/L and 500 mg/L, respectively, whereas no significant interference effects were observed in the presence of nitrite, sulphide, and silicate in the evaluated range (up to 1000 mg/L) (Figure S1). The interference effect associated with both As(V) and silicate has been attributed to the formation of blue-colored products upon reaction with molybdate [31]. It should be highlighted that the reported 3D-µPAD was less prone to the above-mentioned interferences than the 4500P-E standard method, whose analytical response is affected in the presence of As(V) 0.1 mg/L, Cr(VI), and sulphide at 1 mg/L, and nitrite and silicate at 10 mg/L [7]. Additionally, the effect of other potential interferences, such as FeCl_3_, CuCl_2_, CaCl_2_, NaCl, KCl, KNO_3_, NH_4_Cl, Na_2_CO_3_, NaHCO_3_, Na_2_SO_4_, NH_4_VO_3_, and H_3_BO_3_ was studied in the range 1 to 1000 mg/L, and the results are provided in Figures S2 and S3. As can be deduced from the figure, no interference effects were observed, with the only exception of FeCl_3_ at concentration levels equal or higher than 500 mg/L, leading to a slight signal intensity reduction. Fe(III) is also indicated as a potential interference in the 4500-P automated ascorbic acid reduction method [7].

## 4. Analytical Performance and Assessment of GAC Profile

**Figure 8:**
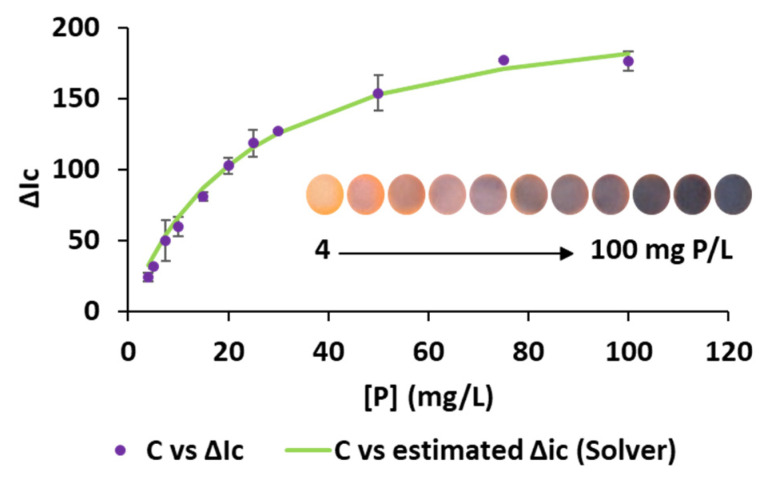
Analytical signal for P (mg P/L) standards of different channels in the red channel experimentally obtained (violet point) and estimated by solver (green line). The inset shows the detection areas of the 3D-µPAD at increasing concentrations of P.

The analytical characteristics of the proposed method were obtained under optimal experimental conditions. A non-linear relationship between the analytical response and phosphate concentration (Figure 8) was observed, in agreement with what has been frequently reported in the literature for colorimetric PADs and µPADs [19,45,46,47]. Experimental data were successfully fitted to a rectangular hyperbolic function (Equation (1)), as proposed elsewhere [48].

(3) $$\Delta I_{c}=\frac{{\Delta I}_{c_{max}}\cdot C}{k+C}$$

where C is the concentration of phosphate, ∆Ic is the analytical response, ΔIc_max_ corresponds to the maximum color intensity that can be obtained, and k is the concentration corresponding to ΔIc_max_/2.

Reorganizing Equation (3), a linear relationship between k/[(∆Ic_max_)/(∆I_c_) − 1)] (i.e., the estimated concentration values) and C was obtained, with a slope of 1.03 ± 0.02 L/mg and intercept of −0.79 ± 0.52 (Figure 8 and Figure S3), close to the ideal values (i.e., 1 and 0, respectively). The ΔIc_max_ and k values that minimize the residual sum of squares were estimated by using the solver tool in Microsoft Excel (Office 2019) and were found to be 225.2 and 23.8 mg/L, respectively.

The limits of detection (LOD) and quantification (LOQ), estimated according to the 3σ and 10σ criteria, were 0.015 mg P/L and 0.05 mg P/L, respectively. The precision of the method, expressed as relative standard deviation (RSD), was evaluated in terms of repeatability and intermediate precision (during 3 consecutive days), being 4.8% (*n* = 8 replicates, 5 mg P/L) and 7.1% (*n* = 24 replicates, 5 mg P/L), respectively.

The degree of compliance of the developed assay with the 12 principles of green analytical chemistry [49] was evaluated using the Analytical GREEnness calculator (AGREE) [50] and compared to that of the standard method for phosphate determination (Method 4500 P-D). As can be deduced from the pictograms shown in Figure S5 of the Supplementary Material, the developed methodology shows a significantly greener profile than that of the standard method, mainly due to the lower sample volume required for the analysis (P2), lower generation of wastes (P7), higher sample throughput (P8), and lower amounts of toxic substances used in the analysis (P9).

### Analysis of Environmental Samples

The applicability of the developed 3D-µPAD to the determination of phosphate in environmental samples, namely waters, soils, and sediments, was evaluated, and the obtained results are shown in Table 1 and Table 2. As can be observed, the analyzed real aqueous samples did not show detectable levels of phosphate, and recoveries ranging from 96 to 106% were obtained. Thus, matrix effects were not observed when dealing with the analysis of aqueous samples of varying complexity. In addition, the colorimetric assay was applied to the determination of phosphorus in an agricultural soil, using 0.5 M NaHCO_3_ as extractant. The results obtained by the analysis of the agricultural soils with the proposed assay were statistically comparable to the values assigned by the organizing entity at a 95% confidence level, since the experimental t value (t_cal_) was lower than the critical t value (t_crit_ 3.18) at this level of significance (Table 2). Additionally, satisfactory z-scores of 0.27 and 0.06 were obtained, thus meeting the acceptance criteria in the proficiency test.

Furthermore, the total P determined in the sewage sludge CRM 029 and the P-extractable content in the BCR 684 sediment were statistically comparable to the certified values.

## 5. Conclusions

A miniaturized colorimetric assay for the determination of phosphate in environmental samples using a 3D-µPAD with smartphone-based readout is reported in this work. The molybdenum blue reaction was the basis for the non-instrumental colorimetric determination of phosphate. Experimental parameters were systematically evaluated for optimal performance, considering geometric aspects of 3D-µPADs, digitization, and image processing parameters, as well as parameters affecting the formation of the colored product. Unlike previously reported PADs and µPADs for phosphate determination, the proposed 3D-µPAD showed acceptable stability when stored under refrigeration, or even at room temperature especially when using ethylene glycol, thus facilitating its applicability for on-site analysis. In addition, the 3D-µPAD enabled the simultaneous and replicated processing of blanks and standards/samples, thus minimizing the uncertainty of the analytical response while increasing the sample throughput. Additionally, the 3D-µPAD showed higher tolerance to potential interferences and was found to be more environmentally benign and affordable than the standard method of analysis for phosphate determination, showing improvements in terms of reagents and samples consumption, waste generation, and portability. Further research on phosphate determination using PADs is expected to focus on the improvement of detection limits through integrated preconcentration steps, while ensuring selectivity for the intended application, enabling multiplex analysis for the simultaneous determination of additional analytes, expanding applicability to a broader variety of samples and advancing device designs toward commercializable formats suitable for on-site analysis.

## Figures and Tables

**Table 1:** Results obtained for the recovery study of aqueous samples.

Sample	Spiked Concentration (mg P/L)	Obtained Concentration (mg P/L) (*n* = 3) ^a^	RSD (%)	Recovery (%)	t_cal_ ^b^
Mineral water	0	<LOD	-	-	-
	5	5.3 ± 0.5	9.4	106 ± 10	1.04
Seawater	0	<LOD	-	-	-
	5	5.0 ± 0.3	6.0	100 ± 7	0.00
River water	0	<LOD	-	-	-
	5	5.1 ± 0.3	5.9	102 ± 6	0.58
Synthetic wastewater I	195.94	195.0 ± 19.5 ^c^	10.0	99.5 ± 10	0.08
Synthetic wastewater II	97.28	93.9 ± 3.3 ^c^	3.5	96.5 ± 3.4	1.77

^a^ Mean value ± standard deviation. ^b^ For a 95% confidence level, the t_crit_ is 4.30 (*n* = 3, 2 freedom degrees, 2 tails). ^c^ These samples were diluted before analysis. *n*, number of replicates.

**Table 2:** Results obtained for the analysis of soil and sediment samples.

Sample	Assigned or Certified Concentration (mg P/kg)	Obtained Concentration (mg P/L) (*n* = 4) ^a^	RSD (%)	Recovery (%)	t_cal_ ^b^
Agricultural soil 1 NaHCO_3_ extractable-P	62 ± 15 ^a^	66 ± 5	6.9	107 ± 7	1.7
Agricultural soil 2 NaHCO_3_ extractable-P	48 ± 16 ^a^	49 ± 4	7.4	102 ± 8	1.8
CRM 029 Soil Total P	21,000 ± 3000 ^c^	19,729 ± 243	1.2	94 ± 1 2.1	2.1
BCR 684 sediment NaOH- extractable-P	550 ± 21 ^c^	541 ± 35	6.5	98 ± 6 1.9	1.9

^a^ Mean value ± standard deviation. ^b^ t_crit_ is 3.18 (*n* = 4, α 0.05, 2 tails). ^c^ Mean value ± expanded uncertainty (coverage factor = 2). *n*, number of replicates.

## Data Availability

Data are contained within the article or Supplementary Material. Data will be made available on request.

## Supplementary Materials

The following supporting information can be downloaded at: https://www.mdpi.com/article/10.3390/s26010335/s1, Figure S1: Evaluation of interferences indicated in the 4500P-E standard method; Figure S2: Evaluation of other potential interferences. (A) NaCl, (B) KCl, (C) FeCl_3_, (D) CuCl_2_, (E) KNO_3_, and (F) NH_4_Cl; Figure S3: Evaluation of other potential interferences. (A) Na_2_CO_3_, (B) NaHCO_3_, (C) Na_2_SO_4_, (D) NH_4_VO_3_, and (E) H_3_BO_3_; Figure S4: (A) Calibration curve for P determination. The orange points represent the fitted values derived from the determination of k and ΔIc_max_ with solver. (B) Plot of the estimated P concentration (mg P/L) vs. P concentration (mg P/L); Figure S5: AGREE pictograms derived from the GAC assessment of the standard method for phosphate determination (A) and the proposed assay (B); Table S1: Colorimetric PADs and µPADs reported in the literature for phosphate determination.

## Abbreviations

The following abbreviations are used in this manuscript:

3D-µPAD	Tridimensional microfluidic paper-based analytical device
12-MPA	Molybdophosphoric acid
AGREE	Analytical GREEnness calculator
APHA	American Public Health Association
CRMs	Certified reference materials
EG	Ethylene glycol
EV	Exposition value
LOD	Limit of detection
LOQ	Limit of quantification
ICP-OES	Inductively coupled plasma optical emission
ICP-MS	Inductively coupled plasma mass spectrometry
ISO	International Standardization Organization
PADs	Paper-based assays
PMB	Phosphomolibdenum blue
pTsOH	p-toluene sulfonic acid
RGB	Red, green, blue
RSD	Relative standard deviation
µPADs	Microfluidic paper-based analytical devices
UV-vis	Ultraviolet–visible
WB	White balance
