# Supplementary material for: A 3D Microfluidic Paper-Based Analytical Device with Smartphone-Based Colorimetric Readout for Phosphate Sensing
Source: Sensors (Basel). 2026 Jan 4;26(1):335. doi: 10.3390/s26010335 (PMC12788290; doi:10.3390/s26010335)
Supplement: Supplementary file 1 [file sensors-26-00335-s001.zip › sensors-4042630-supplementary.pdf]

A 3D microfluidic paper-based analytical device with smartphone-based colorimetric readout for phosphate sensing

J. M. Graña-Dosantos, F. Pena-Pereira, C. Bendicho, I. de la Calle*

Centro de Investigación Mariña, Departamento de Química Analítica e alimentaria, Grupo QA2, Edificio CC Experimentais, Universidade de Vigo, Campus de Vigo, As Lagoas, Marcosende, Vigo, 36310, Spain

*corresponding author: incalle@uvigo.gal

Supplementary Material

Figure S1. Evaluation of interferences indicated in the 4500P-E standard method. A) Si, B) As(V), C) Na_2S, D) K_2CrO_4, and E) $NaNO_2$.

Figure S2. Evaluation of other potential interferences. A) NaCl, B) KCl, C) $FeCl_3$, D) $CuCl_2$, E) KNO_3, and F) NH_4Cl.

Figure S3. Evaluation of other potential interferences. A) Na_2CO_3, B) $NaHCO_3$, C) Na_2SO_4, D) NH_4VO_3, and E) H_3BO_3.

Figure S4. A) Calibration curve for P determination. The orange points represent the fitted values derived from the determination of k and ΔIc_{max} with solver. B) Plot of the estimated P concentration (mg P/L) vs. P concentration (mg P/L).

Figure S5. AGREE pictograms derived from the GAC assessment of the standard method for phosphate determination (A) and the proposed assay (B).

Table S1. Colorimetric PADs and μPADs reported in the literature for phosphate determination.

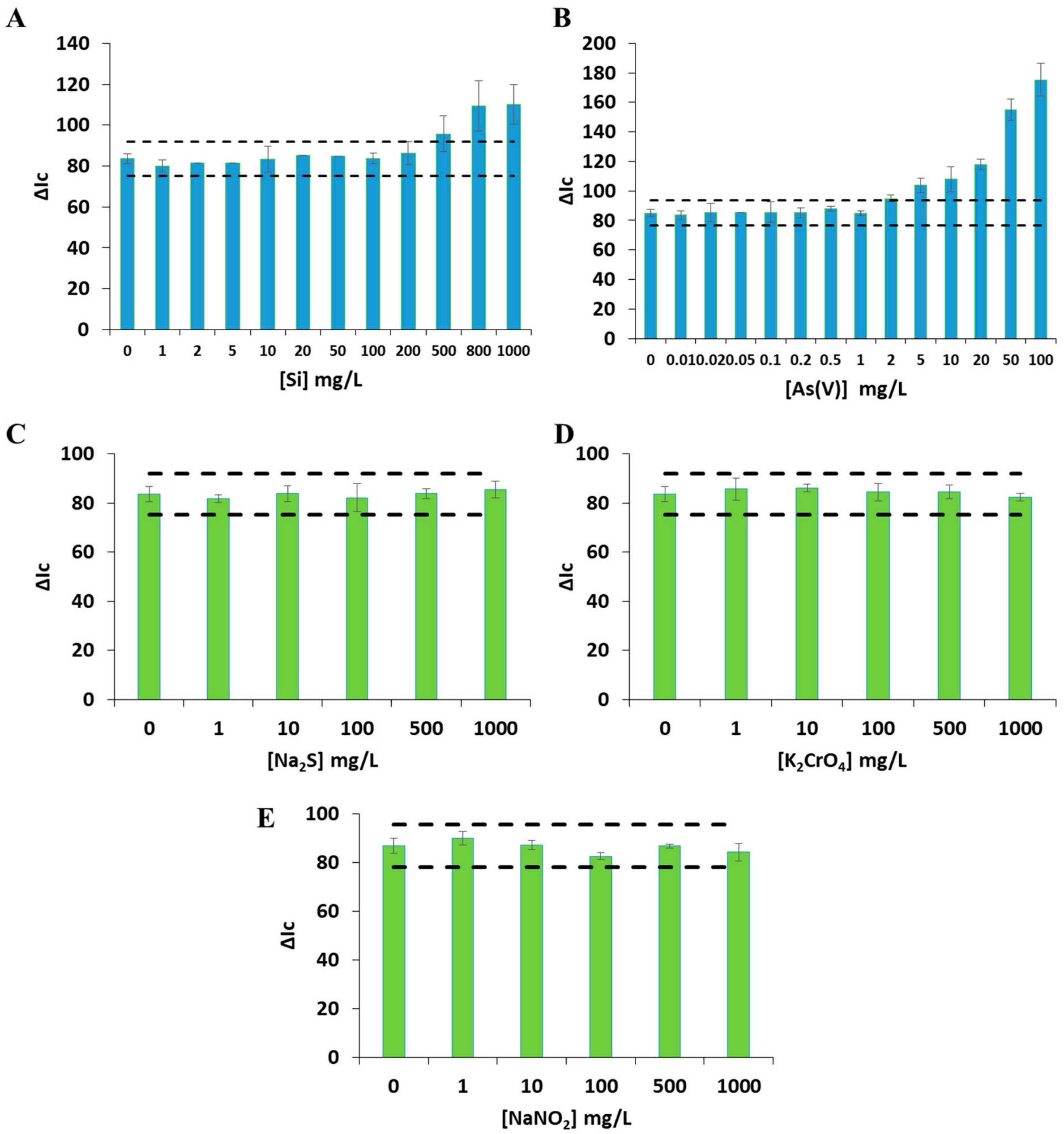

Figure S1. Evaluation of interferences indicated in the 4500P-E standard method. A) Si, B) As(V), C) Na_2S, D) K_2CrO_4, and E) $NaNO_2$.

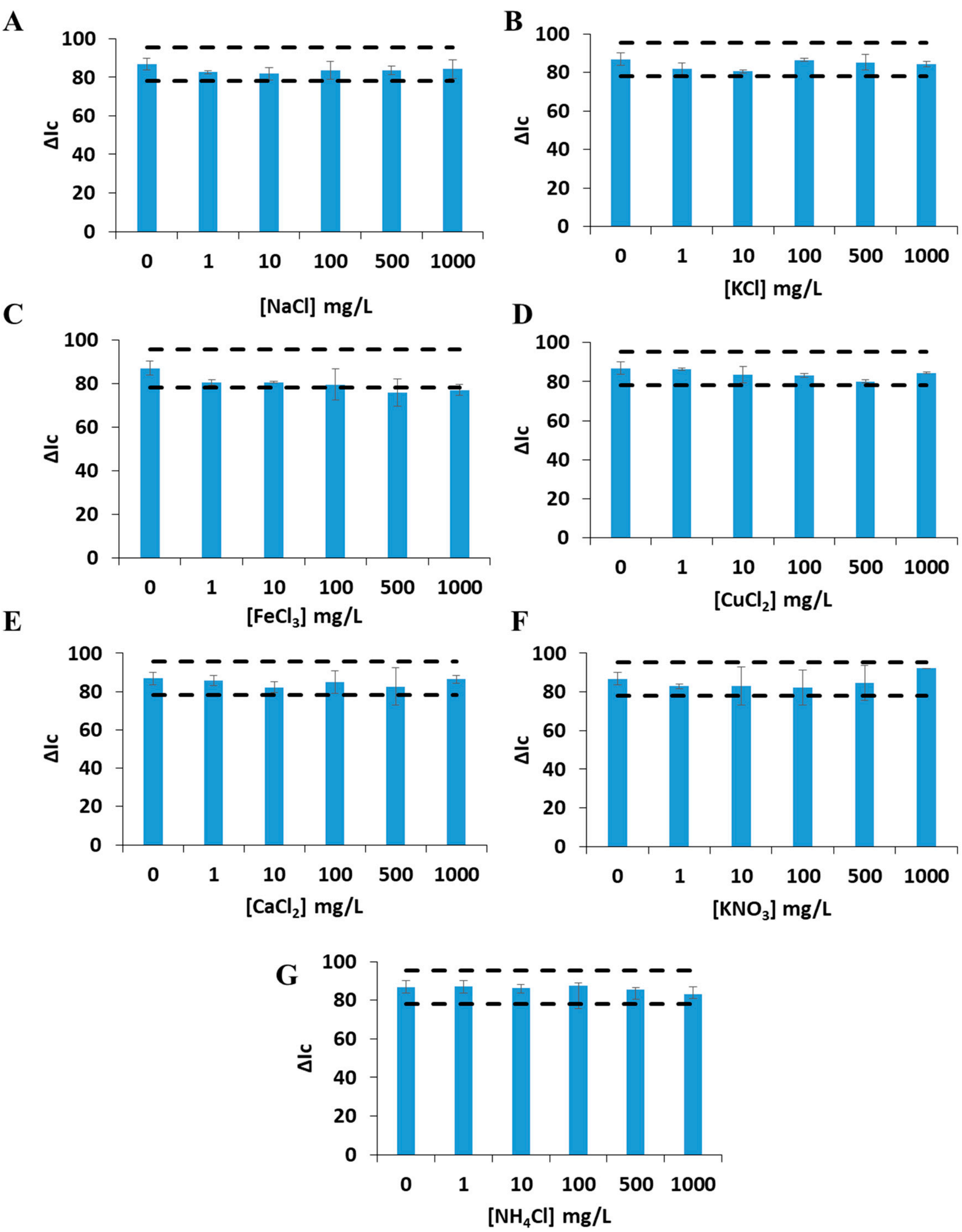

Figure S2. Evaluation of other potential interferences. A) NaCl, B) KCl, C) $FeCl_3$, D) $CuCl_2$, E) KNO_3, and F) NH_4Cl.

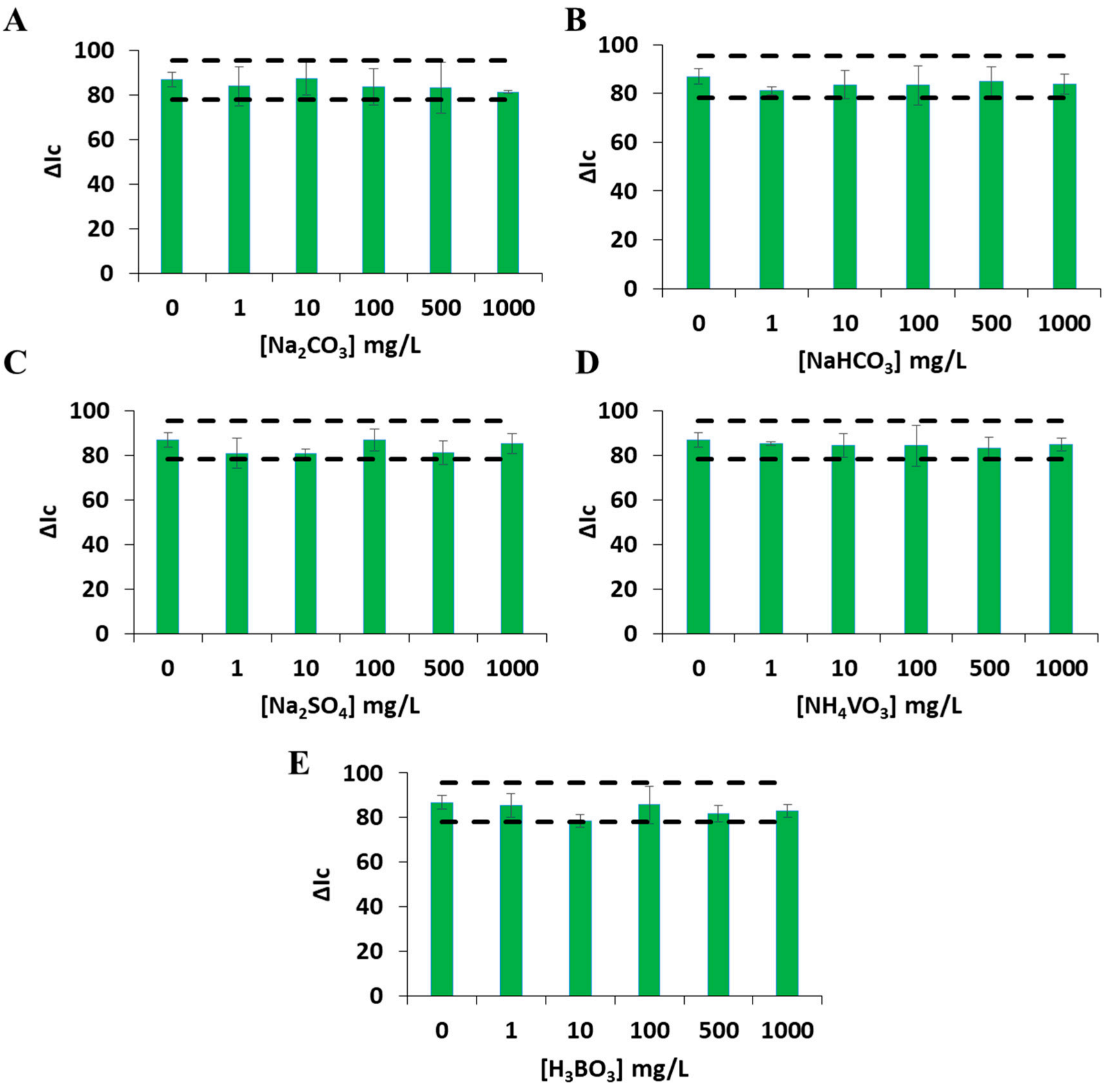

Figure S3. Evaluation of other potential interferences. A) Na_2CO_3, B) $NaHCO_3$, C) Na_2SO_4, D) NH_4VO_3, and E) H_3BO_3.

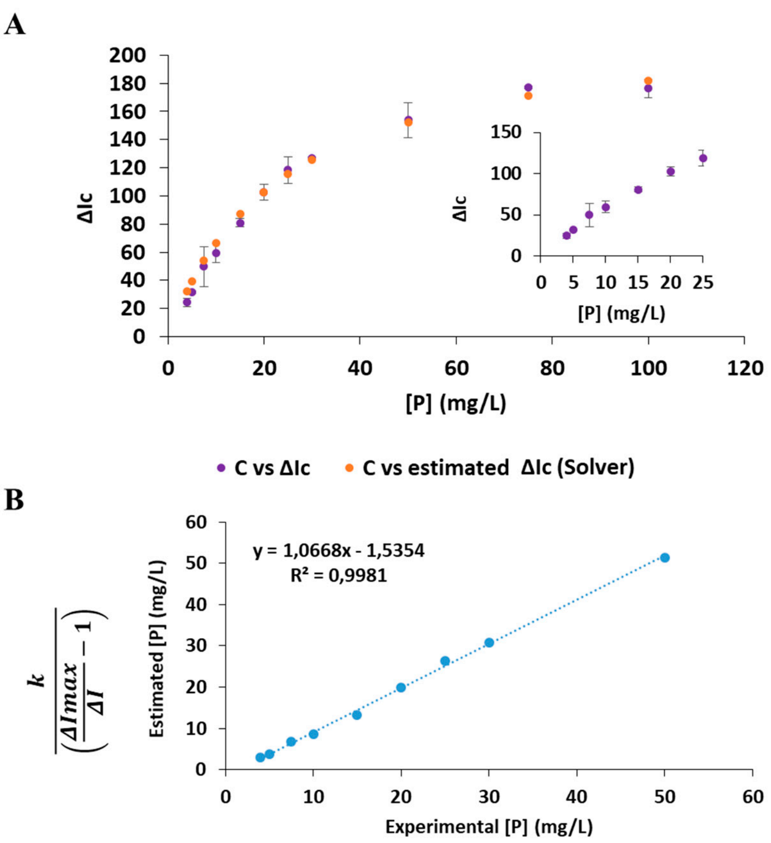

Figure S4. A) Calibration curve for P determination. The orange points represent the fitted values derived from the determination of k and ΔIcmax with solver. B) Plot of the estimated P concentration (mg P/L) vs. P concentration (mg P/L).

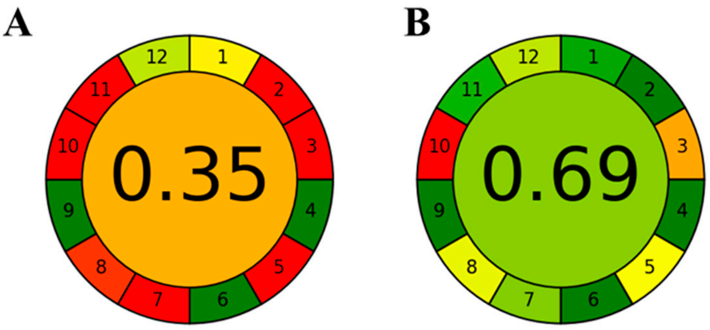

Figure S5. AGREE pictograms derived from the GAC assessment of the standard method for phosphate determination (A) and the proposed assay (B).

Table S1. Colorimetric PADs and µPADs reported in the literature for phosphate determination.

PAD design	Year	Hydrophobization	Reagents and conditions of the PAD	Preloaded PAD/µPAD and other observations	Sample	Working range (mg P/L)	LOD (mg P/L)	RSD (%)	Stability	Reference
2D-µPAD and 3D-µPAD 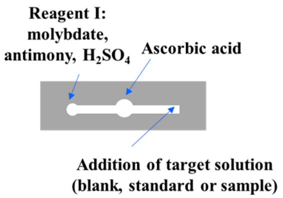 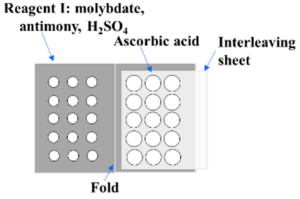	2012	Ink printed with alkenylketene dimer in n-heptane (thermal treatment 150 ºC, 30 min)	Whatman 4<br>77.8 µg of ammonium heptamolybdate<br>323.7 µg of sulfuric acid<br>2 µg of antimony and potassium tartrate<br>132.1 µg ascorbic acid<br>Interleaving sheet for avoiding contact between 2 zones<br>10 µL of sample<br>Reaction time: 40 min	Yes.<br>-Interleaving sheets.<br>-Laminated PAD.<br>-Punch the PAD to add the sample.<br>-Add a drop of the sample.	Surface water, soil water, and seawater	0.2-10	0.05	<2	24 h room temperature,<br>48 h polymeric sheets room temperature,<br>20 days ambient <4 ºC unpunched,<br>112 days -20 ºC unpunched	[1]
3D-µPAD 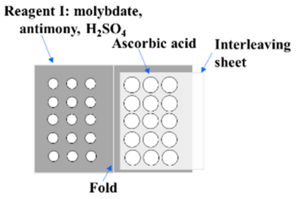	2014	Ink printed with alkenylketene dimer in n-heptane (thermal treatment 150 ºC, 30 min)	Whatman 4<br>77.8 µg of ammonium heptamolybdate<br>323.7 µg of sulfuric acid<br>2 µg of antimony and potassium tartrate<br>132.1 µg ascorbic acid<br>Interleaving sheet for avoiding contact between 2 zones<br>10 µL of sample<br>Reaction time: 10 min	Yes<br>-Interleaving sheets.<br>-Laminated PAD.<br>-Punch the PAD to add the sample.<br>-Add a drop of the sample.	Soil solution	0.1-1 and 1-10	0.05	-	1 day without interleave<br>3 days acetate interleaving sheet,<br>15 days Teflon interleaving sheet	[2]
Spot test 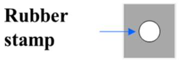	2019	Use of a rubber stamp Polydimethylsiloxane (PDMS): hexane (thermal treatment to cure the PDMS at 80 ºC, 30 min)	0.357 g ammonium metavanadate<br>7.143 g ammonium molybdate in acid	Yes<br>The transparent plastic layer was used to cover the front side of the spot test.<br>-Add a drop of the sample.	Water	326 – 6526	-	-	5 days at room temperature	[3]

2D-µPAD<br>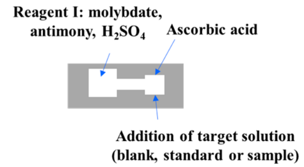 	2020	Wax printed and thermal treatment (120 ºC, 2 min)	Whatman 4<br>2433 µg of ammonium heptamolybdate<br>18390 µg of sulfuric acid<br>62.6 µg of antimony and potassium tartrate<br>2113.4 µg ascorbic acid<br>48694 µg ethyleneglycol<br>25 µL of sample<br>Reaction time: 4 min	Yes<br>-Air drying of each aliquot of ascorbic acid (4 x 3 µL) for 20 min.<br>-Self-adhesive laminated.<br>-Add a drop of the sample.	Seawater, synthetic freshwater	0.03-3.3	0.05	-	250 days (35 weeks) (dark, < 4ºC or -20ºC) with ethyleneglycol<br>1 week room temperature with ethyleneglycol	[4]
Dip-3D-µPAD<br>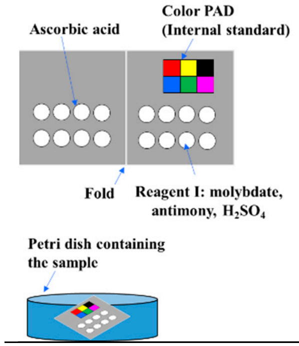 	2021	Wax printed (thermal treatment, 3 times at 125 ºC)	Whatman 1<br>62 µg of ammonium heptamolybdate<br>2 µg of antimony and potassium tartrate<br>8.8 µg ascorbic acid<br>1902 µg p-toluenesulfonic acid<br>Scalpel to do a whole<br>Dip the PAD in a Petri dish containing the sample (20 mL of sample)<br>Reaction time: 3 min	Yes<br>-Reagents are allowed to air dry (30 min) and dried overnight (-20ºC).<br>-Laminated at 80 ºC.<br>-PAD is totally immersed in a Petri dish containing the sample.	River water	0.6-3.3	1	-	28 days (freezer, -20ºC)<br>1 week room temperature	[5]
Dip test strips<br>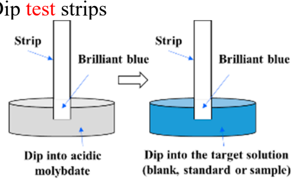 	2021	-	Whatman 1<br>Tween 80 (70 % m/m) (sonication, 10 min)<br>Brilliant green (leave 2 h undisturbed)<br>Strip washed and oven dried at 60 ºC for 30 min.<br>Immersion of strip in ammonium molybdate acidified<br>Reaction time: 5 min	No<br>-Test strip containing brilliant green is immersed into a molybdate acidic solution immediately before sample immersion.	Tap water, treated effluent, lake water	0.3-13.6	0.07	-	90 days	[6]

Dip test strips 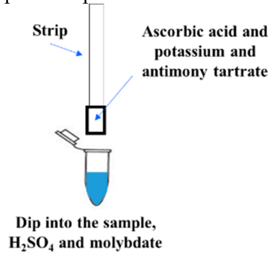	2021	-	Whatman blotting paper<br>Paper modified with 0.5 M ascorbic acid and 6 mM potassium and antimony tartrate.<br>In a vial: 600 µL of sample + 20 µL of 3.6 M sulfuric acid + 40 µL of Mo reagent (0.85 M ammonium heptamolybdate, 0.5 M<br><br><u>Reaction time</u>: 1 min + 3 min	No<br>-Only ascorbic acid and tartrate of antimony and potassium are immobilized in the paper strip (immersion 2 min, and air dry for 2 h)	Seawater	0.03-8.2	0.04	1.8	4 months (expected lifetime: 2 years)	[7]
Test strips (Quantofix and Indigo commercial kit) 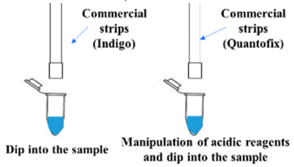	2021	-	Two commercial kits<br><u>Reaction time</u>: 1 min	Yes (Indigo strips)<br>No (Quantofix strips, manipulation of acidic reagents)	Water	0.03-16.3	0.1	<0.5	2 years	[8]
Circular spot test<br>+ preconcentration by repetitive sample addition and drying 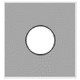	2022	Black marker pen drawn	Whatman 1<br>30.9 µg of ammonium heptamolybdate<br>147.1 µg of sulfuric acid<br>6.1 µg tartaric acid<br>220 µg ascorbic acid<br>2.5 µL of sample<br><u>Reaction time</u>: 5 min	No	Water, wastewater and liquid chemical fertilizer samples	0-100	0.7	3.1	-	[9]
3D-µPAD 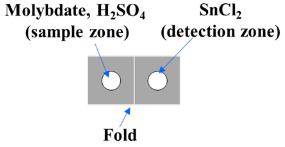	2022	Epoxy resin screen-printed and air drying (3 h).	Whatman 1<br>197.7 µg of ammonium heptamolybdate<br>1961.6 µg of sulfuric acid<br>9954 µg $SnCl_2$<br>10 µL of sample<br><u>Reaction time</u>: 10 min	Yes	Spiked soils	0.16-13	0.08	1-2.7	15 days <2ºC	[10]

2D-µPAD 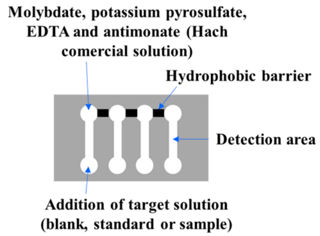	2022	Home-based scan-and-cut printer and assembly into a plastic platform.<br>Permanent marker ink for hydrophobization.	Whatman 1<br>Commercial phosphate reagent sachet (sodium molybdate, potassium pyrosulfate, tetrasodium ethylene diammino tetraacetic acid and antimonite) to dissolve in 3 mL of water<br>Addition of 10 µL of sample and 10 µL of reagent<br><u>Reaction time</u>: 0.5-10 min	No	Solid fertilizer dissolved in water	0-20	0.5	13	-	[11]
DipPAD 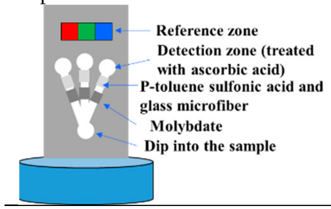	2024	Glass microfiber PAD	1 M p-toluenesulfonic acid<br>19 mM Mo<br>0.01 g/mL of antimony and potassium tartrate<br>10 mM ascorbic acid<br>(Volume not indicated in the article)<br><u>Reaction time</u>: 10-15 min	Yes<br>-Use of double-sided adhesive.<br>-Alternating layers of PET film.	Surface water	0.2-13	0.06	-	1 day at room temperature<br>60 days at room temperature desiccator<br>60 days in a fridge or freezer	[12]
Multiplexed PAD- card (dPAD for phosphate) 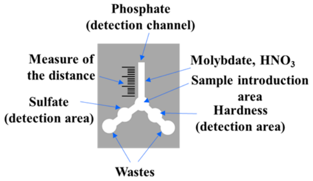	2024	Wax printed (thermal treatment in a hot plate, 150 ºC, 90 s)	49 µg ammonium ortomolybdate $(NH_4)_2MoO_4$<br>189 µg HNO_3<br>13 µL of sample<br><u>Reaction time</u>: 8 min.	Yes<br>-The PAD was taped to the back of the device to prevent leakage.	Water	0.15-31	0.15	-	-	[13]
Multiplexed PAD-flower form Dip dPAD for phosphate) 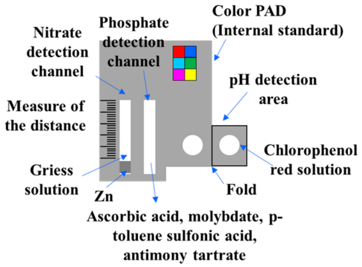	2025	Wax printed (thermal treatment, 125 ºC)	Whatman Grade 4 filter paper<br>556 µg of ammonium heptamolybdate<br>64200 µg of p-toluenesulfonic acid<br>180 µg of antimony and potassium tartrate<br>79.2 µg ascorbic acid	Yes<br>-Laminated between two transparent PET sheets at 125 ºC.	Soil	0.3-7.3	0.9	-	3 days at room temperature<br>12 days refrigerator 4ºC	[14]

µPAD<br>(3D-µPAD and dipPAD) + preconcentration 10-fold<br>(2-layer, longitudinal and transversal transport of sample)<br>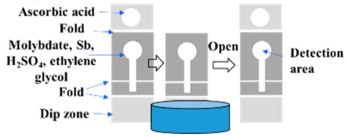	2025	Wax printed (thermal treatment, 120 ºC, 2 min)	Chromatography paper filter 1CHR, Whatman<br>211.3 µg ascorbic acid<br>13 µg of ammonium heptamolybdate<br>98.1 µg of sulfuric acid<br>0.3 µg of antimony and potassium tartrate<br>8.8 µg ascorbic acid<br>259.5 µg Ethylene glycol<br>Reaction time : 3 min (immersion)+ 5 min (reaction)	Yes<br>-Disassembly of the 3D-µPAD and unfolding for detection.	Natural water, soil, and toothpaste	0.05-1 mg/L	0.09 mg/L	4.7 % (intraday)<br>3.0 % (interday)	26 days in a refrigerator	[15]
dPAD + preconcentration (10-fold)<br>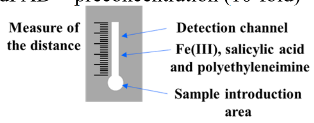	2025	-	Fe(III), Salicylic acid, polyethyleneimine	-	-	1.6-25	0.06	-	-	[16]
3D-µPAD<br>(3-layer, longitudinal and transversal transport of sample)<br>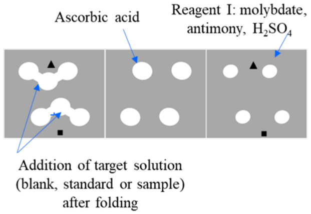	2025	Wax printed (thermal treatment in an oven 130 °C, 2 min)	Whatman 1<br>73 µg of ammonium molybdate<br>362.2 µg of sulfuric acid<br>1.6 µg of antimony and potassium tartrate<br>88.1 µg ascorbic acid<br>Reaction time: 11 min	Yes	Mineral water, seawater, river water, synthetic wastewater, soil extracts, oil digestate	4 - 25	0.02	4.8 - 7.1	45 days freezer,<br>7 days refrigerator,<br>2 days room temperature,<br><br>21 days room temperature (with 1361 µg ethyleneglycol or 1632 µg p-toluenesulfonic acid)	This work

dPAD, distance-based PAD. LOD, limit of detection. PDMS, polydimethylsiloxane. RSD, relative standard deviation. 2D-µPAD, bidimensional microfluidic paper-based device. 3D-µPAD, tridimensional microfluidic paper-based devices.

References

1. Jayawardane, B.M.; McKelvie, I.D.; Kolev, S.D. A Paper-Based Device for Measurement of Reactive Phosphate in Water. *Talanta* **2012**, *100*, 454–460, doi:10.1016/j.talanta.2012.08.021.
2. Jayawardane, B.M.; Wongwilai, W.; Grudpan, K.; Kolev, S.D.; Heaven, M.W.; Nash, D.M.; McKelvie, I.D. Evaluation and Application of a Paper-Based Device for the Determination of Reactive Phosphate in Soil Solution. *J Env. Qual* **2014**, *43*, 1081–1085, doi:10.2134/jeq2013.08.0336.
3. Waghwani, B.; Balpande, S.; Kalambe, J. Development of Microfluidic Paper Based Analytical Device for Detection of Phosphate in Water. *Int. J. Innov. Technol. Explor. Eng. IJITEE* **2019**, *8*, 592–595.
4. Racicot, J.M.; Mako, T.L.; Olivelli, A.; Levine, M. A Paper-Based Device for Ultrasensitive, Colorimetric Phosphate Detection in Seawater. *Sensors* **2020**, *20*, 2766, doi:10.3390/s20102766.
5. Richardson, S.; Iles, A.; Rotchell, J.M.; Charlson, T.; Hanson, A.; Lorch, M.; Pamme, N. Citizen-Led Sampling to Monitor Phosphate Levels in Freshwater Environments Using a Simple Paper Microfluidic Device. *PLOS ONE* **2021**, *16*, e0260102, doi:10.1371/journal.pone.0260102.
6. Choudhary, V.; Philip, L. Stable Paper-Based Colorimetric Sensor for Selective Detection of Phosphate Ion in Aqueous Phase. *Microchem J* **2021**, *171*, 106809, doi:10.1016/j.microc.2021.106809.
7. Heidari-Bafroui, H.; Charbaji, A.; Anagnostopoulos, C.; Faghri, M. A Colorimetric Dip Strip Assay for Detection of Low Concentrations of Phosphate in Seawater. *Sensors* **2021**, *21*, 3125, doi:10.3390/s21093125.
8. Heidari-Bafroui, H.; Ribeiro, B.; Charbaji, A.; Anagnostopoulos, C.; Faghri, M. Portable Infrared Lightbox for Improving the Detection Limits of Paper-Based Phosphate Devices. *Measurement* **2021**, *173*, 108607, doi:10.1016/j.measurement.2020.108607.
9. Phansi, P.; Janthama, S.; Cerdà, V.; Nacapricha, D. Determination of Phosphorus in Water and Chemical Fertilizer Samples Using a Simple Drawing Microfluidic Paper-Based Analytical Device. *Anal. Sci.* **2022**, *38*, 1323–1332, doi:10.1007/s44211-022-00162-y.
10. Thongkam, T.; Hemavibool, K. A Simple Epoxy Resin Screen-Printed Paper-Based Analytical Device for Detection of Phosphate in Soil. *Anal. Methods* **2022**, *14*, 1069–1076, doi:10.1039/D1AY02011K.
11. Kiwfo, K.; Woi, P.M.; Seanjum, C.; Grudpan, K. New Designs of Paper Based Analytical Devices (PADs) for Completing Replication Analysis of a Sample within a Single Run by Employing Smartphone. *Talanta* **2022**, *236*, 122848, doi:10.1016/j.talanta.2021.122848.
12. Aryal, P.; Hefner, C.E.; Martinez, B.; Brack, E.; Henry, C.S. Citizen-Based Water Quality Monitoring: Field Testing a User-Friendly Sensor for Phosphate Detection in Global Surface Waters. *Anal Chem* **2024**, doi:10.1021/acs.analchem.4c02123.
13. Mettakoonpitak, J.; Hatsakhun, P.; Sirasunthorn, N. Alcohol Ink-Modified Microfluidic Paper-Based Analytical Devices for Enhanced White Detection in Simultaneous Determination of Multiple Water Quality Indicators. *Microchim Acta* **2024**, *191*, 680, doi:10.1007/s00604-024-06772-9.
14. Giménez-Gómez, P.; Priem, N.; Richardson, S.; Pamme, N. A Paper-Based Analytical Device for the on-Site Multiplexed Monitoring of Soil Nutrients Extracted with a Cafetière. *Sens Actuators B Chem* **2025**, *424*, 136881, doi:10.1016/j.snb.2024.136881.
15. Danchana, K.; Namba, H.; Kaneta, T. Using a Microfluidic Paper-Based Analytical Device and Solid-Phase Extraction to Determine Phosphate Concentration. *Talanta* **2025**, *295*, 128303, doi:10.1016/j.talanta.2025.128303.
16. Ngamprasertsuk, S.; Duenchay, P.; Dungchai, W. A Simple and Cost-Effective Distance-Based Paper Analytical Device for Phosphate Ion Determination. *Anal Sci* **2025**, *41*, 1365–1372, doi:10.1007/s44211-025-00800-1.
